# Mitogen-Activated Protein Kinase Kinase OsMEK2 Positively Regulates Ca^2+^ Influx and Ferroptotic Cell Death during Rice Immune Responses

**DOI:** 10.3390/antiox13081013

**Published:** 2024-08-20

**Authors:** Juan Wang, Nam Khoa Nguyen, Dongping Liu, Nam-Soo Jwa

**Affiliations:** Division of Integrative Bioscience and Biotechnology, College of Life Sciences, Sejong University, Seoul 05006, Republic of Korea; gloria0826@163.com (J.W.); khoanam050295@gmail.com (N.K.N.); liudongping921@163.com (D.L.)

**Keywords:** MAPK, Ca^2+^ influx, H_2_O_2_, rice, *Magnaporthe oryzae*, ferroptotic cell death

## Abstract

Mitogen-activated protein (MAP) kinase (MAPK) signaling pathway is important in plant immune responses, involved in iron- and reactive oxygen species (ROS)-dependent ferroptotic cell death mediated by Ca^2+^. High Ca^2+^ influx triggered iron-dependent ROS accumulation, lipid peroxidation, and subsequent hypersensitive response (HR) cell death in rice (*Oryza sativa*). Apoplastic Ca^2+^ chelation by EGTA during avirulent *Magnaporthe oryzae* infection altered Ca^2+^, ROS, and Fe^2+^ accumulation, increasing rice susceptibility to infection. By contrast, acibenzolar-*S*-methyl (ASM), a plant defense activator, significantly enhanced Ca^2+^ influx, and H_2_O_2_ accumulation, triggering rice ferroptotic cell death during virulent *Magnaporthe oryzae* infection. Here, we report a novel role of the MAPK signaling pathway in regulating cytoplasmic Ca^2+^ increase during ferroptotic cell death in rice immunity, using the *ΔOsmek2* knockout mutant rice. The knockout of rice *OsMEK2* impaired the ROS accumulation, lipid peroxidation, and iron accumulation during avirulent *M. oryzae* infection. This study has shown that OsMEK2 could positively regulate iron- and ROS-dependent ferroptotic cell death in rice by modulating the expression of *OsNADP-ME*, *OsRBOHB*, *OsPLC*, and *OsCNGC*. This modulation indicates a possible mechanism for how OsMEK2 participates in Ca^2+^ regulation in rice ferroptotic cell death, suggesting its broader role in plant immune responses in response to *M. oryzae* infection.

## 1. Introduction

Plants have evolved numerous defense mechanisms to protect themselves from pathogen attacks [1]. The defense response starts with the successful recognition of pathogens. Pattern recognition receptors (PRRs) recognize pathogen-associated molecular patterns (PAMPs) to activate pattern-triggered immunity (PTI), and intracellular or extracellular receptors which detect effectors from the pathogens to activate effector-triggered immunity (ETI) [2,3]. Mitogen-activated protein (MAP) kinase (MAPK) signaling pathway is one of the major signal pathways involved in intracellular events, such as developmental changes and immune responses. Activation of MAPKs by PRRs induces a series of defense responses, including expression of pathogenesis-related (PR) genes, and plant cell death during plant immune responses [4,5]. One well-studied *Arabidopsis* MAPK cascade in plant immunity is the MEKK1-MKK1/2-MPK4 cascade [6]. MEKK1 interacts with MKK1/2 on the plasma membrane, and MKK1/2 interacts with MPK4 on both the plasma membrane and the nucleus, indicating that the MEKK1-MKK1/2-MPK4 cascade transmits the signal from the PRR to the nucleus [6]. MKK1 and MKK2 function redundantly with MPK4 and MEKK1 to regulate plant immunity by modulating ROS accumulation and plant cell death [6]. Similarly, the *Pseudomonas syringae* effector HopAI1 is an effector that can directly target *Arabidopsis* MPK3 and MAPK6 to inactivate MAPKs to promote pathogenesis [7]. The MAPK cascade plays an essential role in plant innate immunity, involved in both PTI and ETI.

Plant MAPK signaling proceeds through a kinase cascade: MAPK kinase kinase (MAPKKK), MAPK kinase (MAPKK, or MEK), and MAP kinase (MAPK) [8]. One structural non-canonical MAPKK, MPKK10.2, was found to enhance rice resistance against *Xanthomonas oryzae* pv. *oryzicola* infection and drought stress, by phosphorylating MPK6 and MPK3, indicating a role of MAPKK in plant immunity [9]. We previously performed Y2H screening of the rice MAPK interactors and found that OsMEK2 could interact with OsMPK1 and OsMPK6 [10]. This was confirmed by mapping the MAPK interactome network to underline the cellular and physiological responses of the MAPK signaling pathway in rice [11]. When signals are perceived on the plasma membrane by PRRs or NLRs, the MAPK cascade can be activated. MAP kinase kinases (MEKs) regulate transcriptional reprograming by activating MAP kinase (MAPK) [12]. Rice OsMEK2 interacts with OsMPK1, and then OsMPK1 interacts with the downstream transcription factor OsWRKY90 to activate plant immune responses [10,11,13].

The hypersensitive response (HR) in rice has been identified to share characteristics of animal ferroptotic cell death, including iron accumulation, GSH depletion, and lipid peroxidation during avirulent *Magnaporthe oryzae* (*M. oryzae*) infection [14,15]. Ca^2+^ channels and Ca^2+^ signals are well known to be extensively involved in plant immunity [16]; however, the discovery of the HOPZ-ACTIVATED RESISTANCE 1 (ZAR1) resistosome has shown that the role of Ca^2+^ in plant immune responses may be more complex than previously thought [17]. The knockout of rice *OsMEK2* impaired the ROS accumulation, lipid peroxidation, and iron accumulation during avirulent *M. oryzae* infection, indicating that OsMEK2 participates in rice ferroptosis during incompatible rice–*M. oryzae* interaction [13]. Further study has elucidated that OsMEK2 could positively regulate ROS- and iron-dependent ferroptotic cell death in rice, by modulating the expression of rice *NADP-malic enzyme* (*OsNADP-ME*), and *respiratory burst oxidative homologs* (*OsRbohs*), which are plant nicotinamide adenine dinucleotide phosphate (NADPH) oxidases [13]. In rice, Rbohs are localized in the plasma membrane with six transmembrane domains, playing an important role in ROS production during defense signaling [18].

In addition to ROS accumulation, recent studies have found that resistosome-mediated Ca^2+^ influx is an essential feature during plant HR cell death [19,20]. In contrast to the active research on the role of MAPKs in regulating ROS generation [21], MAPK studies on intracellular Ca^2+^ regulation are still limited. In this study, we investigated the role of OsMEK2 in association with Ca^2+^ influx during avirulent *M. oryzae*-induced hypersensitive cell death. We applied the Ca^2+^ chelator ethylene glycol-bis (2-aminoethylether)-*N*, *N*, *N′*, *N′*-tetra-acetic acid (EGTA), and systemic acquired resistance (SAR) inducer acibenzolar-*S*-methyl (ASM) to analyze the role of OsMEK2 in Ca^2+^ regulation during *M. oryzae*–rice interaction. In the present study, we further confirmed that the expression levels of *OsNADP-ME* and *OsRBOHB*, which are important for ROS production, were severely reduced in *ΔOsmek2* rice during avirulent *M. oryzae* infection. Here, we suggest a possible mechanism for how OsMEK2 participates in Ca^2+^ regulation in rice ferroptotic cell death and immune responses.

## 2. Materials and Methods

### 2.1. Plant Materials and Growth Conditions

Seeds of rice (*Oryza sativa* L.) cultivar Donjin (DJ) and Kitaake were obtained from the National Institute of Crop Science (http://www.nics.go.kr, accessed on 1 September 2010), and *ΔOsmek2* mutant seeds were provided by the Rice Functional Genomic Express Database (RiceGE) managed by the Salk Institute (http://signal.salk.edu./cgi-bin/RiceGE, accessed on 8 March 2017). The plants were grown in Baroker soil (Seoul Bio, Seoul, Korea) in a growth chamber and the settings were as follows: 28 °C, 60% humidity, white fluorescent light (150 µmol photons m^−2^ s^−1^), and a 16 h day/8 h night photoperiod.

### 2.2. Fungal Cultures and Growth Conditions

Virulent *M. oryzae* strain PO6-6 and RO1-1 and avirulent strain 007 were obtained from the Center for Fungal Genetic Resources, Seoul National University, Seoul, Korea (http://genebank.snu.ac.kr, accessed on 5 January 2010). *M. oryzae* strains were grown on rice bran agar media (20 g rice bran, 20 g sucrose, and 20 g agar in 1 L water) in the dark at 25 °C for 10 days, followed by sporulation under continuous fluorescent light (80 µmol photons m^−2^ s^−1^) for another 3–4 days. Conidial suspensions were adjusted to 4 × 10^5^ spore mL^−1^ in 0.025% (*v*/*v*) Tween 20 before use [14].

### 2.3. Fungal Inoculation and Infection Evolution

*M. oryzae* conidial suspensions were inoculated onto 6-week-old rice leaf sheaths, and the samples were kept in a dark moistened chamber, at 28 °C, for 48 h. The epidermal 2–3 cell layers were monitored and investigated under a microscope (Zeiss equipped with Axioplan 2, Bentonville, AR, USA). Infected epidermal cells were classified into three infection types: IH (invasive hyphae colonized inside the cell), type Ⅰ HR (severe hypersensitive response), and type Ⅱ HR (mild hypersensitive response). The experiment was independently repeated three times.

### 2.4. Treatment of Pharmacological Compounds

Ethylene glycol-bis (2-aminoethylether)-*N*, *N*, *N′*, *N′*-tetra-acetic acid (EGTA; Sigma-Aldrich, St. Louis, MO, USA) was applied during avirulent *M. oryzae* infection to chelate apoplastic Ca^2+^, and acibenzolar-*S*-methyl (ASM) was applied during virulent *M. oryzae* infection to enhance Ca^2+^ influx [15]. EGTA (3 mM) was treated to rice sheath cells, together with *M. oryzae* 007; ASM (500 µM) was treated to rice sheath cells, 2 h before virulent *M. oryzae* PO6-6 treatment.

### 2.5. Detection and Quantification of Ca^2+^ and H_2_O_2_

Fluo-5F AM is a Ca^2+^ indicator, which monitors intracellular Ca^2+^ [22]. Peroxy orange 1 (PO1) is an H_2_O_2_ probe, which emits orange intracellular fluorescence after incubation with H_2_O_2_ [23]. We applied Fluo-5F AM and PO1 to observe intracellular Ca^2+^ and H_2_O_2_, respectively. Briefly, the leaf sheath epidermal cells were incubated in 50 µM of Fluo-5F AM and 5 µM of PO1 at 37 °C for 1 h, followed by a three-time washing using 3DW. The samples were monitored and investigated under a fluorescence microscope (Zeiss equipped with Axioplan 2). The settings for filters were as follows: green fluorescence: Ex/Em: 450–490/515–565 nm, and red fluorescence: Ex/Em: 546/590 nm.

At least three regions of interest (ROIs) were chosen to quantify the green fluorescence intensities to monitor changes in Ca^2+^ levels at different time points using ImageJ software, https://imagej.net/ij/ (Classic, Wayne Rasband, Bethesda, MD, USA) [24]. To properly present the images which were corresponded with the generated plots, an image enhancement process was carried out when necessary. Especially, in figure in below, the brightness of the green field (GF) and red field (RF) images were enhanced by 50% to compare the changes in cytoplasmic Ca^2+^ and H_2_O_2_ accumulations at different time points. Corrected total cell fluorescence (CTCF) values were calculated as previously described [25,26]: CTCF = integrated fluorescence density − (ROI area × mean fluorescence of background readings). H_2_O_2_ levels were quantified by measuring red fluorescence intensities in a similar way using the ImageJ software (Classic, Wayne Rasband, Bethesda, MD, USA).

### 2.6. Prussian Blue Staining

Prussian blue staining was performed to visualize ferric ion (Fe^3+^) accumulation in rice leaf sheath cells infected with *M. oryzae* [14]. Epidermal layers of rice sheath cells were submerged in the Prussian blue solution (7% potassium ferrocyanide and 2% hydrochloric acid) overnight at room temperature (RT), and the stained samples were washed several times using 3DW to remove excess background. The samples were monitored and observed under a microscope (Zeiss equipped with Axioplan 2).

### 2.7. Glutathione Measurement

Total glutathione (oxidized form and reduced form) and reduced glutathione (GSH) were measured spectrophotometrically [14]. Avirulent *M. oryzae* 007 spores were inoculated onto rice leaf sheaths, and the samples were incubated at 25 °C for 48 h in the dark condition. The samples were ground in liquid nitrogen. An equal amount of the sample powder was mixed with 5% (*w*/*v*) meta-phosphoric acid (Sigma Aldrich) and the samples were centrifuged at 21,000× *g* for 20 min at 4 °C. The supernatants were used immediately to quantify glutathione contents [14]. Reduced glutathione (GSH) can be directly measured by observing OD_412_ after reacting with 5,5′-dithiobis (2-nitrobenzoic acid) (DTNB), and total glutathione (GSSG and GSH) can be measured by reducing GSSG to GSH first, and then measuring OD_412_ after reacting with DTNB [27].

### 2.8. MDA Quantification

Malondialdehyde (MDA) is a product of unsaturated fatty acid peroxidation and can indicate the level of lipid peroxidation in rice leaf sheath samples, using thiobarbituric acid (TBA). Rice leaf sheaths were ground in liquid nitrogen, and the sample powder was mixed with the reaction solution (0.5% [*w*/*v*] TBA [Sigma-Aldrich], 20% [*v*/*v*] trichloroacetic acid [TCA; Sigma-Aldrich], and 0.25 mL of 175 mM NaCl in a total of 2 mL of 50 mM Tris-Cl [pH 8.0]). The samples were kept in a water bath (100 °C) for 5 min and the supernatant was collected by centrifuging at 14,000× *g* for 5 min at 4 °C. The absorbance was measured at 450, 532, and 600 nm with a spectrophotometer (Woongki Science, Seoul, Republic of Korea) [14]. MDA concentration was calculated based on the equation as follows [14].

$$C_{MDA}=\left[6.45\times \left({OD}_{532}-{OD}_{600}\right)\right]-\left(0.56\times {OD}_{450}\right)$$

where *C_MDA_* is the concentration of MDA, and *OD*_450_, *OD*_532_, and *OD*_600_ represent the optical density (*OD*) at 450, 532, and 600 nm, respectively.

### 2.9. RNA Extraction and Gene Expression Analysis

Total RNA was extracted from rice plants using the TRIzol Reagent (Invitrogen) and used for cDNA synthesis. Transcript levels of *OsNADP-ME*, *OsRBOHB*, *OsPLC2*, *OsPLC4*, *OsCNGC2*, *OsCNGC13*, and *OsUbiquitin* (*OsUbi*) genes were analyzed by reverse transcription polymerase chain reaction (RT-PCR) and real-time quantitative RT-PCR (real-time qRT-PCR) (Supplementary Table S1). Transcript levels of genes were normalized relative to that of *OsUbi* and presented as mean ± standard deviation (SD) of three biological replicates.

## 3. Results

### 3.1. OsMEK2 Positively Regulates Cytoplasmic Ca^2+^ and H_2_O_2_ Levels during Avirulent M. oryzae Infection

**Figure 1:**
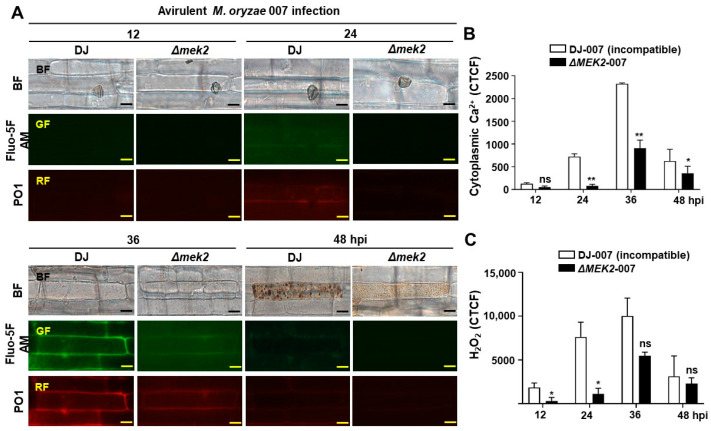
Time-course detection of cytoplasmic Ca^2+^ and H_2_O_2_ accumulation in WT rice DJ and *ΔOsmek2* mutant rice during avirulent *Magnaporthe oryzae* 007 infection. (**A**) Images of cytoplasmic Ca^2+^ staining by Fluo-5F AM and H_2_O_2_ staining by peroxy orange 1 (PO1) in wildtype (WT) rice DJ and *ΔOsmek2* mutant rice at 12, 24, 36, and 48 hpi, during avirulent *M. oryzae* 007 infection. Bars = 10 μm. (**B**) Quantification of cytoplasmic Ca^2+^ accumulation in rice sheath cells infected with *M. oryzae* 007 at 12, 24, 36, and 48 hpi. (**C**) Quantification of cytoplasmic H_2_O_2_ accumulations in rice sheath cells infected with *M. oryzae* 007 at 12, 24, 36, and 48 hpi. Values are means ± SD (*n* = 3 biological repeats). Asterisks above the bar indicated significantly different means, as determined by the ANOVA test (mixed-effects analysis) (* *p* < 0.05, ** *p* < 0.01; ns, not significant). SD, standard deviation.

OsMEK2 is required for ROS, iron accumulation, and lipid peroxidation to cause rice ferroptotic cell death [13]. We investigated cytoplasmic Ca^2+^ and H_2_O_2_ accumulation in rice DJ and *ΔOsmek2* mutant rice during avirulent *M. oryzae* infection at different time points, and the results showed that cytoplasmic Ca^2+^ and H_2_O_2_ were highly accumulated in WT rice DJ during avirulent *M. oryzae* 007 infection at 36 hpi, compared to that in the *ΔOsmek2* mutant rice (Figure 1). Changes in cytoplasmic Ca^2+^ and H_2_O_2_ were analyzed at 12, 24, 36, and 48 hpi in rice DJ and *ΔOsmek2* mutant rice during avirulent *M. oryzae* infection (Figure 1A). In WT rice DJ, cytoplasmic Ca^2+^ and H_2_O_2_ accumulation started from 12–24 hpi and peaked at 36 hpi, however, cytoplasmic Ca^2+^ and H_2_O_2_ accumulation in *ΔOsmek2* mutant rice was significantly impaired from 12–36 hpi (Figure 1). The *ΔOsmek2* mutant rice showed no expression of the *OsMEK2* (Supplementary Figure S1), and had a different disease phenotype during avirulent *M. oryzae* infection, compared to that in the WT rice DJ (Supplementary Figure S2). We classified the disease phenotypes into IH (invasive hyphae grew inside the rice cell), Type I HR (typical hypersensitive cell death with brown granules filled inside the infected rice cell), and Type II HR (mild hypersensitive cell death with light brown color inside the infected rice cell) (Supplementary Figure S2A). The *ΔOsmek2* mutant rice showed a significantly higher HR cell death than WT rice DJ, with the majority of HR cell death being mild HR cell death (Supplementary Figure S2B). To investigate the effects of virulent *M. oryzae* on cytoplasmic Ca^2+^ and H_2_O_2_ accumulation, we inoculated virulent *M. oryzae* PO6-6 onto WT rice DJ and *ΔOsmek2* mutant rice. Virulent *M. oryzae* infection induced minor cytoplasmic Ca^2+^ and H_2_O_2_ accumulation in both WT rice DJ and *ΔOsmek2* mutant rice, with no significant difference between WT rice DJ and *ΔOsmek2* mutant rice (figure in below).

### 3.2. EGTA Abrogates Ca^2+^, ROS Increase, Iron Accumulation, and HR Cell Death in WT and ΔOsmek2 Mutant Rice

**Figure 2:**
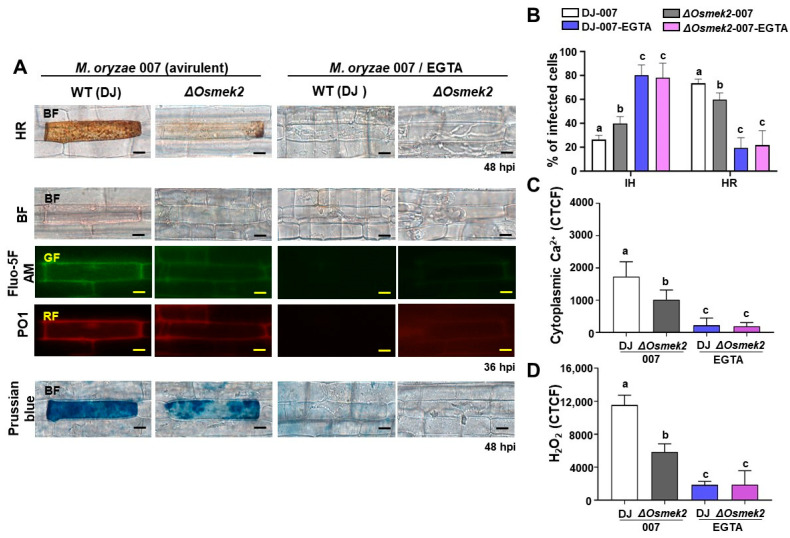
Effects of EGTA on HR cell death, cytoplasmic Ca^2+^, H_2_O_2_ accumulation, and iron accumulation in WT rice DJ and *ΔOsmek2* mutant rice during avirulent *Magnaporthe oryzae* 007 infection. (**A**) Images of HR cell death, cytoplasmic Ca^2+^ staining, H_2_O_2_ staining, and iron staining in wildtype (WT) rice DJ and *ΔOsmek2* mutant rice. Bars = 10 μm. (**B**) Quantification of different infection types in rice sheath cells of wildtype (WT) rice DJ and *ΔOsmek2* mutant rice. IH, invasive hyphae; HR, hypersensitive response. (**C**) Quantification of cytoplasmic Ca^2+^ accumulation in rice sheath cells of wildtype (WT) rice DJ and *ΔOsmek2* mutant rice. (**D**) Quantification of cytoplasmic H_2_O_2_ accumulation in rice sheath cells of wildtype (WT) rice DJ and *ΔOsmek2* mutant rice. Values are means ± SD (*n* = 3 biological repeats). Different letters above the bar indicated significantly different means, as determined by one-way ANOVA test followed by Tukey’s HSD. SD, standard deviation.

During avirulent *M. oryzae* infection, cytoplasmic Ca^2+^ elevation, ROS accumulation, and iron accumulation induced rice ferroptotic cell death in WT rice DJ (Figure 2). Avirulent *M. oryzae* infection induced high levels of cytoplasmic Ca^2+^ elevation, and ROS and iron accumulation in rice leaf sheath cells, as visualized by the microscopy (Figure 2A). We compared cytoplasmic Ca^2+^, ROS accumulation, iron accumulation, and HR cell death in rice DJ and *ΔOsmek2* mutant rice during avirulent *M. oryzae* inoculation and EGTA treatment to investigate whether Ca^2+^ influx is required for rice ferroptotic cell death. To determine the proper concentration of EGTA, we applied 1 and 3 mM EGTA to investigate the effects on HR cell death, based on our primary experimental data. It was shown that 3 mM EGTA greatly suppressed HR cell death, with a better effect on reducing HR cell death than 1 mM EGTA treatment (Supplementary Figure S3). During avirulent *M. oryzae* infection, WT rice DJ showed a typical HR cell death (48 hpi), featured by a high cytoplasmatic Ca^2+^ concentration (36 hpi), ROS accumulation (36 hpi), and iron accumulation (48 hpi) (Figure 2). However, the *ΔOsmek2* mutant rice showed less HR cell death but more cell infections; especially, the HR cell death that occurred in *ΔOsmek2* mutant rice was less dense, compared with that which occurred in the WT rice DJ (Figure 2). Also, less cytoplasmatic Ca^2+^ and less ROS accumulation (36 hpi) were detected in the *ΔOsmek2* mutant rice. In addition, the *ΔOsmek2* mutant rice had less iron accumulation compared to that in the WT rice DJ during avirulent *M. oryzae* infection (Figure 2). The treatment of the Ca^2+^ chelator EGTA blocked the Ca^2+^ influx, ROS accumulation, iron accumulation, and eventual cell death (Figure 2).

### 3.3. ASM Enhanced Ca^2+^, ROS Increase, Iron Accumulation, and HR Cell Death in WT and ΔOsmek2 Mutant Rice

**Figure 3:**
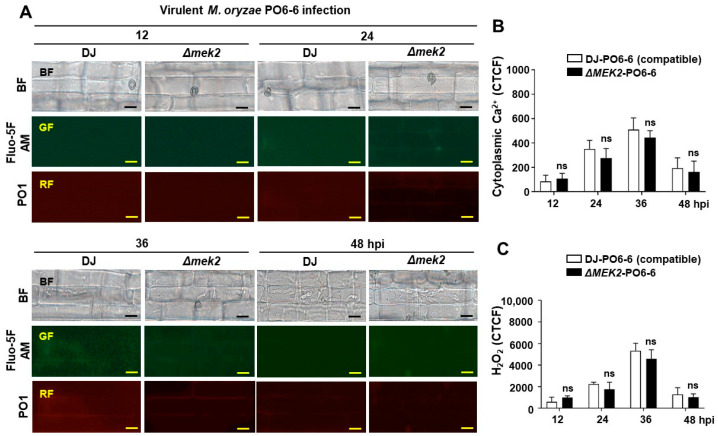
Time-course detection of cytoplasmic Ca^2+^ and H_2_O_2_ accumulation in WT rice DJ and *ΔOsmek2* mutant rice during virulent *Magnaporthe oryzae* PO6-6 infection. (**A**) Images of cytoplasmic Ca^2+^ staining by Fluo-5F AM and H_2_O_2_ staining by peroxy orange 1 (PO1) in wildtype (WT) rice DJ and *ΔOsmek2* mutant rice at 12, 24, 36, and 48 hpi, during virulent *M. oryzae* PO6-6 infection. Bars = 10 μm. (**B**) Quantification of cytoplasmic Ca^2+^ accumulation in rice sheath cells infected with *M. oryzae* PO6-6 at 12, 24, 36, and 48 hpi. (**C**) Quantification of cytoplasmic H_2_O_2_ accumulations in rice sheath cells infected with *M. oryzae* PO6-6 at 12, 24, 36, and 48 hpi. Values are means ± SD (*n* = 3 biological repeats). Data were analyzed by the ANOVA test (mixed-effects analysis) (ns, not significant). SD, standard deviation.

**Figure 4:**
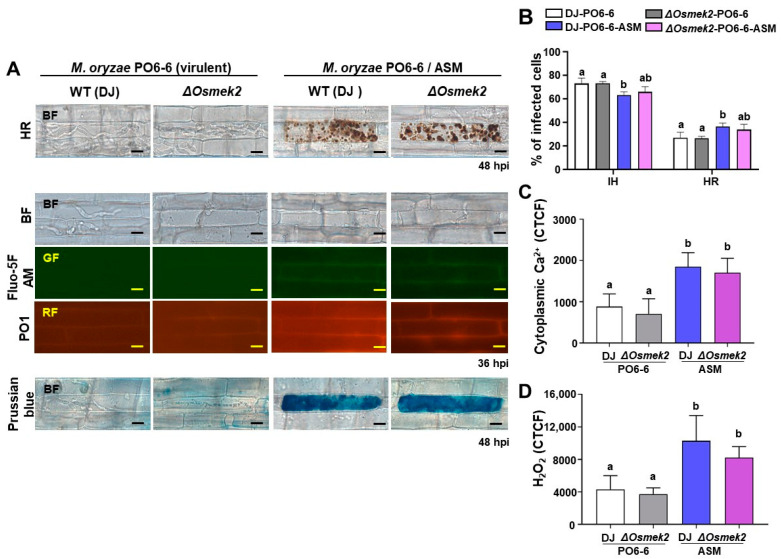
Effects of ASM on HR cell death, cytoplasmic Ca^2+^, H_2_O_2_ accumulation, and iron accumulation in WT rice DJ and *ΔOsmek2* mutant rice during virulent *Magnaporthe oryzae* PO6-6 infection. (**A**) Images of HR cell death, cytoplasmic Ca^2+^ staining, H_2_O_2_ staining, and iron staining in wildtype (WT) rice DJ and *ΔOsmek2* mutant rice. Bars = 10 μm. (**B**) Quantification of different infection types in rice sheath cells of wildtype (WT) rice DJ and *ΔOsmek2* mutant rice. IH, invasive hyphae; HR, hypersensitive response. (**C**) Quantification of cytoplasmic Ca^2+^ accumulation in rice sheath cells of wildtype (WT) rice DJ and *ΔOsmek2* mutant rice. (**D**) Quantification of cytoplasmic H_2_O_2_ accumulation in rice sheath cells of wildtype (WT) rice DJ and *ΔOsmek2* mutant rice. Values are means ± SD (*n* = 3 biological repeats). Different letters above the bar indicated significantly different means, as determined by one-way ANOVA test followed by Tukey’s HSD. SD, standard deviation.

During virulent *M. oryzae* infection, cytoplasmic Ca^2+^ and ROS levels were at baseline in WT rice DJ, with invasive hyphae growing inside the rice sheath cells, as visualized by the microscopy (Figure 3A). We compared cytoplasmic Ca^2+^, ROS accumulation, iron accumulation, and HR cell death in rice DJ and *ΔOsmek2* mutant rice during virulent *M. oryzae* inoculation and ASM treatment to investigate whether Ca^2+^ influx is required for rice ferroptotic cell death. To determine the proper concentration of ASM, we applied 125 and 500 µM ASM to investigate the effects on HR cell death, based on our primary experimental data. Consequently, 500 µM ASM significantly increased HR cell death, with a better effect on inducing HR cell death than 125 µM ASM treatment (Supplementary Figure S4). During virulent *M. oryzae* infection, both WT rice DJ and the *ΔOsmek2* mutant rice showed a susceptible phenotype, with fungal hyphae growing inside the rice epidermal cells (Figure 4A). The treatment of the SAR inducer ASM enhanced the Ca^2+^ influx (36 hpi), ROS accumulation (36 hpi), iron accumulation (48 hpi), and HR cell death (48 hpi) in both WT rice DJ and the *ΔOsmek2* mutant rice (Figure 4). However, the *ΔOsmek2* mutant rice showed less HR cell death after ASM treatment, compared to that in ASM-treated WT rice DJ (Figure 4B).

### 3.4. OsMEK2 Increased Glutathione Content and Impaired Lipid Peroxidation during Avirulent M. oryzae Infection

**Figure 5:**
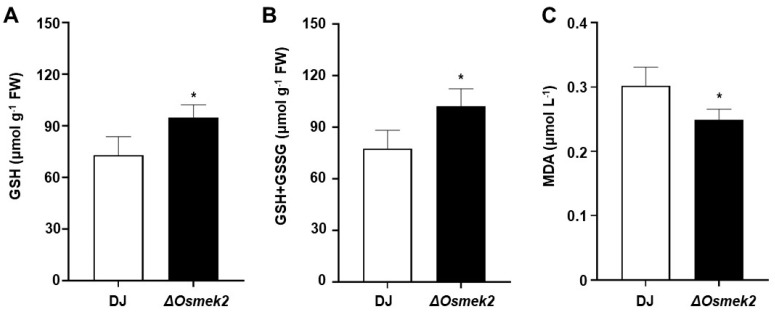
Comparisons of GSH depletion and lipid peroxidation in WT rice DJ and *ΔOsmek2* mutant rice during avirulent *Magnaporthe oryzae* 007 infection. (**A**) Quantification of reduced glutathione (GSH) in rice leaf sheaths in WT rice DJ and *ΔOsmek2* mutant rice at 48 hpi. Values are means ± SD (*n* = 3) of GSH contents. (**B**) Quantification of total glutathione (GSH + GSSG) in rice leaf sheaths in WT rice DJ and *ΔOsmek2* mutant rice at 48 hpi. Values are means ± SD (*n* = 3) of total glutathione contents. (**C**) Determination of lipid peroxidation by quantifying malondialdehyde (MDA) in rice leaf sheaths in WT rice DJ and *ΔOsmek2* mutant rice at 48 hpi. Values are means ± SD (*n* = 3) of MDA concentrations. Asterisks above the bar indicated significantly different means, as determined by the Student’s *t*-test (* *p* < 0.05). SD, standard deviation.

Rice ferroptotic cell death is featured with glutathione depletion and lipid peroxidation [14]. The glutathione content in the cell reflects the redox level of the cell and represents the ROS scavenging capacity of the cell [28]. To compare the glutathione content and ROS scavenging capacity in WT rice DJ and the *ΔOsmek2* mutant rice during avirulent *M. oryzae* 007 infection, we investigated reduced glutathione (GSH) and total glutathione (GSSG + GSH), and the results showed that *ΔOsmek2* mutant rice had higher GSH, as well as total glutathione content, representing a higher capacity for ROS scavenging (Figure 5A,B).

ROS accumulation always leads to oxidative stress that finally results in MDA formation, and the reaction of ROS and lipids can cause lipid peroxidation [29]. To compare the MDA content and the lipid peroxidation level in WT rice DJ and the *ΔOsmek2* mutant rice during avirulent *M. oryzae* 007 infection, we measured the MDA content, and the results showed that the *ΔOsmek2* mutant rice showed less MDA content, compared to WT rice DJ, suggesting a lower lipid peroxidation level (Figure 5C).

### 3.5. Defense-Related Genes Were Downregulated in the ΔOsmek2 Mutant Rice during Avirulent M. oryzae Infection

**Figure 6:**
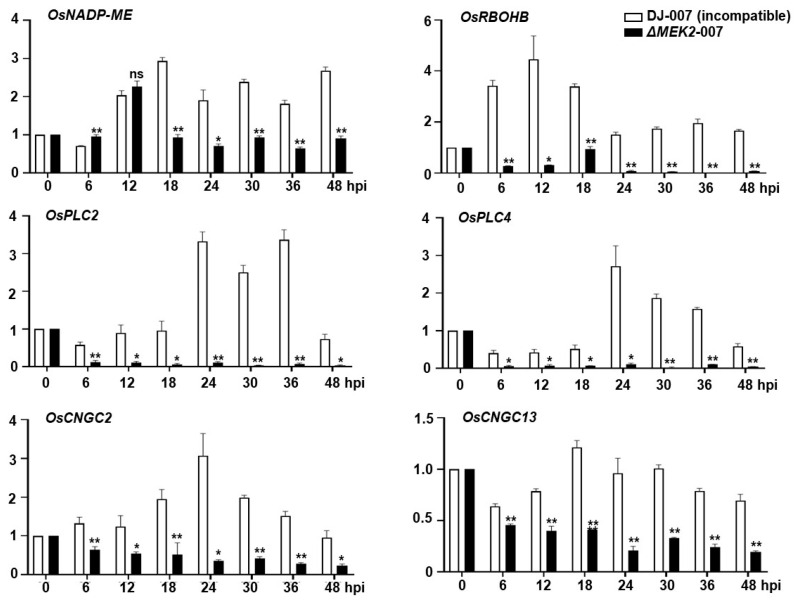
Real-time reverse transcription (qRT-PCR) analysis of time-course expressions of ROS- and calcium-related genes in leaf sheaths of rice DJ and *ΔOsmek2* during avirulent *Magnaporthe oryzae* 007 infection. Relative expression levels of *OsNADP-ME*, *OsRBOHB*, *OsPLC2*, *OsPLC4*, *OsCNGC2*, and *OsCNGC13* were normalized by the expression of *OsUbiquitin* (*OsUbi*). The data represent means ± SD of relative gene expression levels in rice leaf sheaths from three independent experiments. Asterisks above the bars indicate significant differences, as determined by the ANOVA test (mixed-effects analysis) (* *p* < 0.05, ** *p* < 0.01; ns, not significant). SD, standard deviation.

To analyze the ROS regulation and cellular oxidative condition, we analyzed the expression levels of *OsNADP-ME* and *OsRBOHB* at different time points after avirulent *M. oryzae* inoculation to rice sheath cells of WT and *ΔOsmek2* mutant rice, and the results showed that the *ΔOsmek2* mutant rice had lower expressions of *OsNADP-ME* and *OsRBOHB*, compared to that in the WT rice DJ. The lower expressions of *OsNADP-ME* and *OsRBOHB* corresponded to the lower level of cellular ROS in the *ΔOsmek2* mutant rice (Figure 6).

We analyzed the expressions of *OsPLC2* and *OsPLC4*, *OsCNGC2* and *OsCNGC13*, and the results showed that the expressions of *OsPLC2* and *OsPLC4*, *OsCNGC2* and *OsCNGC13* were significantly suppressed in *ΔOsmek2* mutant rice during avirulent *M. oryzae* infection, compared to those in the WT rice DJ, suggesting that the external and internal Ca^2+^ release were less active in the *ΔOsmek2* mutant rice, where less HR cell death occurred (Figure 6).

## 4. Discussion

### 4.1. OsMEK2 Regulated the Accumulation of Cytoplasmic Ca^2+^ and H_2_O_2_ Induced by Avirulent M. oryzae

Avirulent *M. oryzae* induces cytoplasmic Ca^2+^ increase and ROS accumulation to trigger immune responses [14,30]. Increased cytoplasmic Ca^2+^ can come from the apoplast or the internal stores [31]. Apoplastic Ca^2+^ acts as the main supplier of pathogen-induced cytoplasmic Ca^2+^ influx [32]. When plant cells were treated with elicitors, radioactive Ca^2+^ uptake from the apoplast could be observed, suggesting that apoplastic Ca^2+^ could be transported to the cytoplasm and participate in cellular activities during plant immunity [33,34]. The inhibition of Ca^2+^ influx compromised MAPK activation, suggesting that Ca^2+^ influx is necessary for the MAPK signaling pathway [35]. A sustained cytoplasmic Ca^2+^ elevation could activate sustained MAPK signaling, which could ultimately lead to HR cell death [36]. The constitutive expression of tobacco MEK2 or Arabidopsis MEK4/5 could induce H_2_O_2_ production, indicating the importance of the MAPK cascades during ROS production and plant immune responses [37]. However, MAPK cascades are not the sole signaling pathway to regulate plant HR cell death. This study investigated cytoplasmic Ca^2+^ and H_2_O_2_ accumulation in both WT rice DJ and the *ΔOsmek2* mutant rice during avirulent *M. oryzae* infection. The *ΔOsmek2* mutant rice showed impaired cytoplasmic Ca^2+^ and H_2_O_2_ accumulation (Figure 1), suggesting that OsMEK2 is required for cytoplasmic Ca^2+^ increase and ROS accumulation for inducing rice ferroptotic cell death. During plant immunity, there is extensive interplay between Ca^2+^ and ROS [38]. Ca^2+^ is extensively involved in plant immune responses for signaling transduction [39]. Recently, the discovery of resistosomes has suggested a potential new role for Ca^2+^ in plant HR cell death [19]. Specifically, the elevation of cytoplasmic Ca^2+^ has emerged as a determinant of cell death during the plant immune responses [19]. Cytoplasmic Ca^2+^ increase can be due to the influx from apoplast through cyclic-nucleotide-gated channels (CNGC) or from the internal stores through the phospholipase C (PLC) pathway [40,41]. It is worth investigating the Ca^2+^ and ROS homeostasis in rice DJ and *ΔOsmek2* mutant rice to elucidate the interplay between Ca^2+^, ROS, and the MAPK signaling pathway during rice immune responses.

### 4.2. EGTA Blocked Ca^2+^ Influx, ROS Accumulation, Iron Accumulation, and HR Cell Death in WT and ΔOsmek2 Mutant Rice

Ethylene glycol-bis (2-aminoethylether)-*N*, *N*, *N′*, *N′*-tetra-acetic acid (EGTA) could effectively chelate Ca^2+^ influx in tobacco during the bacteria-induced hypersensitive response [42]. Moreover, EGTA also significantly decreased cell death induced by INF1, suggesting that Ca^2+^ is an essential component during elicitin-induced cell death [43]. The recent discovery of resisitosomes has highlighted the importance of apoplastic Ca^2+^ during plant ferroptotic cell death [44]. In this study, we used EGTA as an apoplastic Ca^2+^ chelator to investigate the role of apoplastic Ca^2+^ during avirulent *M. oryzae*-induced rice ferroptotic cell death. EGTA treatment completely blocked Ca^2+^ influx, ROS accumulation, iron accumulation, and HR cell death in both WT and *ΔOsmek2* mutant rice (Figure 2), suggesting that the influx of apoplastic Ca^2+^ into the cytosol might be an early essential event to trigger downstream rice immune responses against *M. oryzae* infection. Rice cells infected with avirulent *M. oryzae* accumulate numerous dark brown granules during hypersensitive cell death response while simultaneously restricting fungal hyphae growth [14]. However, cheating the apoplastic Ca^2+^ using EGTA completely prohibited the accumulation of such granules, with the invasive hyphae extensively growing inside the rice cell (Figure 2A). Glutathione depletion, ROS accumulation, lipid peroxidation, and iron accumulation are marker events during rice ferroptotic cell death [14], and this study has suggested that Ca^2+^ increase might be an earlier essential event during rice hypersensitive response. EGTA could block Ca^2+^ influx from the apoplast to the cytoplasm, and this prevented the subsequent ROS accumulation and lipid peroxidation, suggesting that Ca^2+^ plays a vital role in ROS generation and downstream defense signaling.

### 4.3. ASM Enhanced Ca^2+^ Influx, ROS Accumulation, Iron Accumulation, and HR Cell Death in WT and ΔOsmek2 Mutant Rice

Acibenzolar-*S*-methyl (ASM) is one of the salicylic acid (SA) analogs that protects rice against various pathogens [45]. Pretreatment of ASM on apple seedlings could induce resistance against fire blight caused by *Erwinia amylovora*, by inducing the accumulation of defense-related enzymes such as peroxidases and β-1,3-glucanases [46]. In tomato seedlings, the application of ASM effectively reduced disease severity and induced plant resistance by increasing the activity of peroxidase (POX) and chitinase [47]. Cheng et al. [48] applied both ASM and MAKP inhibitor PD98059 to investigate the effects of ASM on the role of the MAPK cascade on apple resistance against *Penicillium ex pansum*. ASM treatment inhibited lesion expansion, promoted ROS accumulation, and regulated the expression of the genes involved in the MAPK cascade, including MdMAPK2, MdMAPK4, MdMAPKK1, and MdMAPK3 [48]. In this study, we investigated the effects of the plant activator ASM on rice ferroptotic cell death. ASM induced Ca^2+^ influx, ROS accumulation and iron accumulation, and the consequent HR cell death in WT and *ΔOsmek2* mutant rice, indicating a role of ASM in cytoplasmic Ca^2+^ increase, and the subsequent immune responses during rice ferroptotic cell death (Figure 4). Surprisingly, ASM induced a significant increase in intracellular Ca^2+^ levels even in *ΔOsmek2* mutant rice. Although ASM induced slightly less HR cell death in *ΔOsmek2* mutant rice compared to WT rice DJ (Figure 4B), the maintenance of ferroptotic cell death in *ΔOsmek2* mutant rice suggests the possibility that ASM primarily regulates Ca^2+^ through mechanisms other than MAPK signaling. To investigate whether ASM triggers external or internal Ca^2+^ influx to the cytosol, we used ASM/EGTA co-treatment. Compared to ASM treatment, ASM/EGTA co-treatment resulted in less cytoplasmic Ca^2+^ accumulation and HR cell death (Supplementary Figure S5). This suggests that ASM-induced cytoplasmic Ca^2+^ elevation may have primarily originated from the apoplastic Ca^2+^ influx. However, further research is still needed to elucidate the detail mechanism by which ASM mediates the increase in intracellular Ca^2+^ in rice cells.

### 4.4. OsMEK2 Regulated Glutathione Depletion and Impaired Lipid Peroxidation during Avirulent M. oryzae Infection

Glutathione acts as a crucial antioxidant to control reactive oxygen species (ROS) during plant immune responses [49]. GSH depletion could directly cause ROS accumulation due to its antioxidant function [50]. Glutathione depletion has been identified as a hallmark event during ferroptosis and other forms of cell death [51]. At normal conditions, around 90% of glutathione exists in the reduced form (GSH), and 10% exists in the oxidized form (GSSG) [52]. When the plants encounter biotic or abiotic stresses, cellular oxidative stress changes the ratio of glutathione into the oxidized form [52,53]. We compared the levels of reduced glutathione (GSH) and total glutathione (GSH + GSSG) in WT rice DJ and the *ΔOsmek2* mutant rice during avirulent *M. oryzae* 007 infection, and the higher glutathione contents in the *ΔOsmek2* mutant rice support the lower H_2_O_2_ levels and the reduced HR cell death (Figure 1 and Figure 2). Glutathione depletion during avirulent *M. oryzae* infection can induce H_2_O_2_ accumulation, which might lead to iron accumulation and lipid peroxidation, ultimately causing ferroptotic cell death [54].

Lipid peroxides (ROOH) are an important class of reactive oxygen species, and lipid peroxidation has been identified as a key downstream feature of ferroptotic cell death [55]. Lipids are essential for maintaining the integrity of cell membranes, and the peroxidation of lipids could alter the structure, properties, or dynamics of the cell membranes [56]. Malondialdehyde (MDA), a biomarker for lipid peroxidation in biological samples, is one well-described lipid peroxide degradation product, and the reaction of MDA with thiobarbituric acid could yield a product that can be detected by the spectrophotometer [14,57]. The MDA content in the *ΔOsmek2* mutant rice was less compared to that in the WT rice DJ (Figure 5C), suggesting less peroxidized lipids of the cellular membrane.

### 4.5. Time-Course Expression of Defense-Related Genes in WT and ΔOsmek2 Mutant Rice during M. oryzae Infection

Respiratory burst oxidase homologue (RBOH), the nicotinamide adenine dinucleotide phosphate (NADPH) oxidase, could generate superoxide ions in a Ca^2+^-dependent manner, which are later converted into H_2_O_2_ by superoxide dismutase (SOD), inducing a subsequent Ca^2+^ influx and cytoplasmic Ca^2+^ elevation [36]. During pathogen infections, OsRBOHB plays a vital role in ROS burst and rice immune responses [58]. The NADPH conversation contributes to the acidification in the cytoplasm, modulating plant immune responses [59]. MAPK cascades regulate transcriptional reprograming via the WRKY transcription factors [60]. Our earlier research has discovered that OsMEK2 could interact with OsMPK1, which moves from the cytoplasm to the nucleus and interacts with the transcription factor OsWRKY90 [10,11,13]. The NADP-malic enzyme (NADP-ME) functions to catalyze the oxidative decarboxylation of malate under stress conditions [61]. In the current research, we found that the *NADP-malic enzyme* (*OsNADP-ME*) and *NADP-oxidase respiratory burst oxidase homolog protein B* (*OsRBOHB*) were downregulated in the *ΔOsmek2* mutant rice during avirulent *M. oryzae* 007 infection (Figure 6), suggesting an impaired ability of the mutant rice for ROS accumulation. Cytoplasmic Ca^2+^ could activate the RBOHB, both directly and indirectly [62,63]. In the *ΔOsmek2* mutant rice, less cytoplasmic Ca^2+^ was detected (Figure 1), which may lead to the downregulation of *OsRBOHB* (Figure 6). The phospholipase C (PLC) can cleave the membrane phospholipid phosphoatidylinositol-4,5-bisphopshate (PIP_2_) to produce inositol trisphosphate (IP_3_), which enables the Ca^2+^ release from the internal stores [41]. While the membrane-bound cyclic nucleotide-gated channels (CNGCs) facilitate Ca^2+^ influx to the cytoplasm from the apoplast, increasing the cytoplasmic Ca^2+^ concentration [40]. The current data showed that *OsPLC2* and *OsPLC4*, and *OsCNGC2* and *OsCNGC13* were downregulated in the *ΔOsmek2* mutant rice during avirulent *M. oryzae* 007 infection, suggesting an impaired ability of the mutant rice for cytoplasmic Ca^2+^ increase from both internal and external Ca^2+^ release (Figure 6). The qRT-PCR data suggest that OsMEK2 might contribute to rice ferroptotic cell death by regulating ROS- and Ca^2+^-related genes to modulate ROS accumulation and cytoplasmic Ca^2+^ elevation.

### 4.6. Model of OsMEK2-Mediated Rice Ferroptotic Cell Death and Plant Immune Response

**Figure 7:**
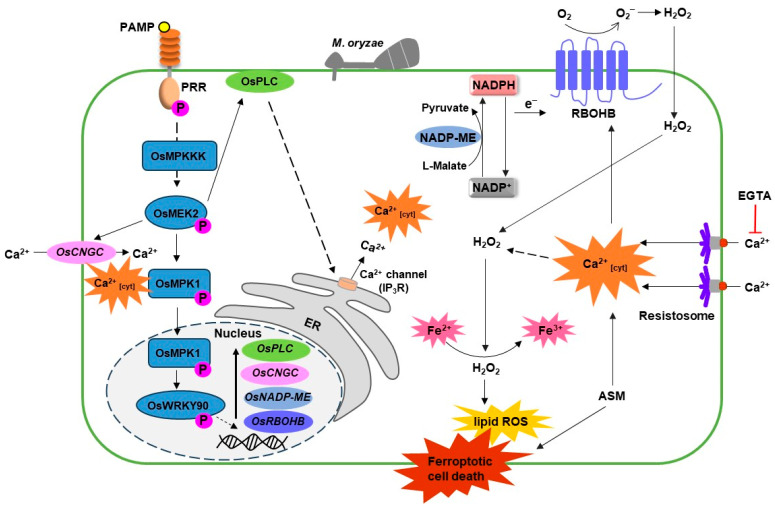
Model of OsMEK2-mediated rice ferroptotic cell death and plant immune response. Membrane-bound PRRs recognize the *M. oryzae* effector and activate the mitogen-activated protein kinase (MAPK) pathway. Activated OsMEK2 triggers OsMPK1-OsWRKY90 pathway in the nucleus, upregulating the *NADP-malic enzyme* (*OsNADP-ME*), *NADP-oxidase* (*OsRBOHB*), *phospholipase C* (*OsPLC*), and *cyclic nucleotide-gated channels* (*OsCNGC*). OsNADP-ME and OsRBOHB regulate cellular ROS production, and OsPLC and OsCNGC facilitate internal (stores such as ER) and external (apoplast) Ca^2+^ influx to the cytosol, playing important roles during rice ferroptotic cell death. Resistosomes form calcium-permeable channels which enable Ca^2+^ influx to mediate cell death, while EGTA chelates apoplastic Ca^2+^, preventing Ca^2+^ influx and subsequent cell death. The plant activator ASM stimulates cytoplasmic Ca^2+^ increase through a yet undescribed mechanism, contributing to ferroptotic cell death. Solid arrows and solid T-shaped lines indicate positive and negative regulators, respectively. Dotted arrows indicate indirect or unverified connections.

Based on the previous publications and the current data, we proposed a model of the OsMEK2-mediated signaling pathway and its involvement in rice ferroptotic cell death (Figure 7). The mitogen-activated protein kinase (MAPK) pathway is activated through the recognition of the *M. oryzae* effector by PRRs. In the MAPK signaling cascade, OsMEK2 triggers the OsMPK1-OsWRKY90 pathway in the nucleus, upregulating *OsNADP-ME*, *NADP-oxidase* (*OsRBOHB*), *phospholipase C* (*OsPLC*), and *cyclic nucleotide-gated channels* (*OsCNGC*). NADP-ME converts L-malate to pyruvate, with NADP+ being converted to NADPH. NADPH gives electrons to RBOHB to promote ROS production. Ca^2+^ release from the internal stores and Ca^2+^ influx through the cell membrane are regulated by the PLC signaling pathway and the CNGC channels. The produced ROS and elevated cytoplasmic Ca^2+^ regulate the downstream immune responses, including iron accumulation and rice ferroptotic cell death. The recently discovered resistosomes are localized on the plasma membrane to facilitate apoplastic Ca^2+^ influx to mediate rice ferroptotic cell death, while EGTA can chelate apoplastic Ca^2+^ to prevent Ca^2+^ influx, as well as the subsequent ROS accumulation, iron accumulation, and the eventual cell death. The plant activator ASM used in this the study stimulates cytoplasmic Ca^2+^ increase and ROS accumulation through an unelucidated mechanism, contributing to ferroptotic cell death. Further research on the detail mechanism of ASM in the increase of intracellular Ca^2+^ is necessary to understand ferroptotic cell death and plant immune responses.

## Data Availability

Data are contained within the article.

## Supplementary Materials

The following supporting information can be downloaded at https://www.mdpi.com/article/10.3390/antiox13081013/s1. Figure S1: Genotyping and transcriptional analyses of *ΔOsmek2* mutant rice. Figure S2: Disease phenotypes of *ΔOsmek2* mutants after inoculation with avirulent *Magnaporthe oryzae*. Figure S3: Effects of EGTA on HR cell death in wildtype rice DJ after inoculation with avirulent *Magnaporthe oryzae*. Figure S4: Effects of ASM on HR cell death in wildtype rice DJ after inoculation with virulent *Magnaporthe oryzae*. Table S1: Primers used in this study. Figure S5: Effects of EGTA on ASM-mediated cytoplasmic Ca^2+^ influx and HR cell death during virulent *M. oryzae* infection.
