# Supplementary material for: Mitogen-Activated Protein Kinase Kinase OsMEK2 Positively Regulates Ca2+ Influx and Ferroptotic Cell Death during Rice Immune Responses
Source: Antioxidants (Basel). 2024 Aug 20;13(8):1013. doi: 10.3390/antiox13081013 (PMC11351908; doi:10.3390/antiox13081013)
Supplement: Supplementary file 1 [file antioxidants-13-01013-s001.zip › Figure S1-S5.pptx]

## Slide 1
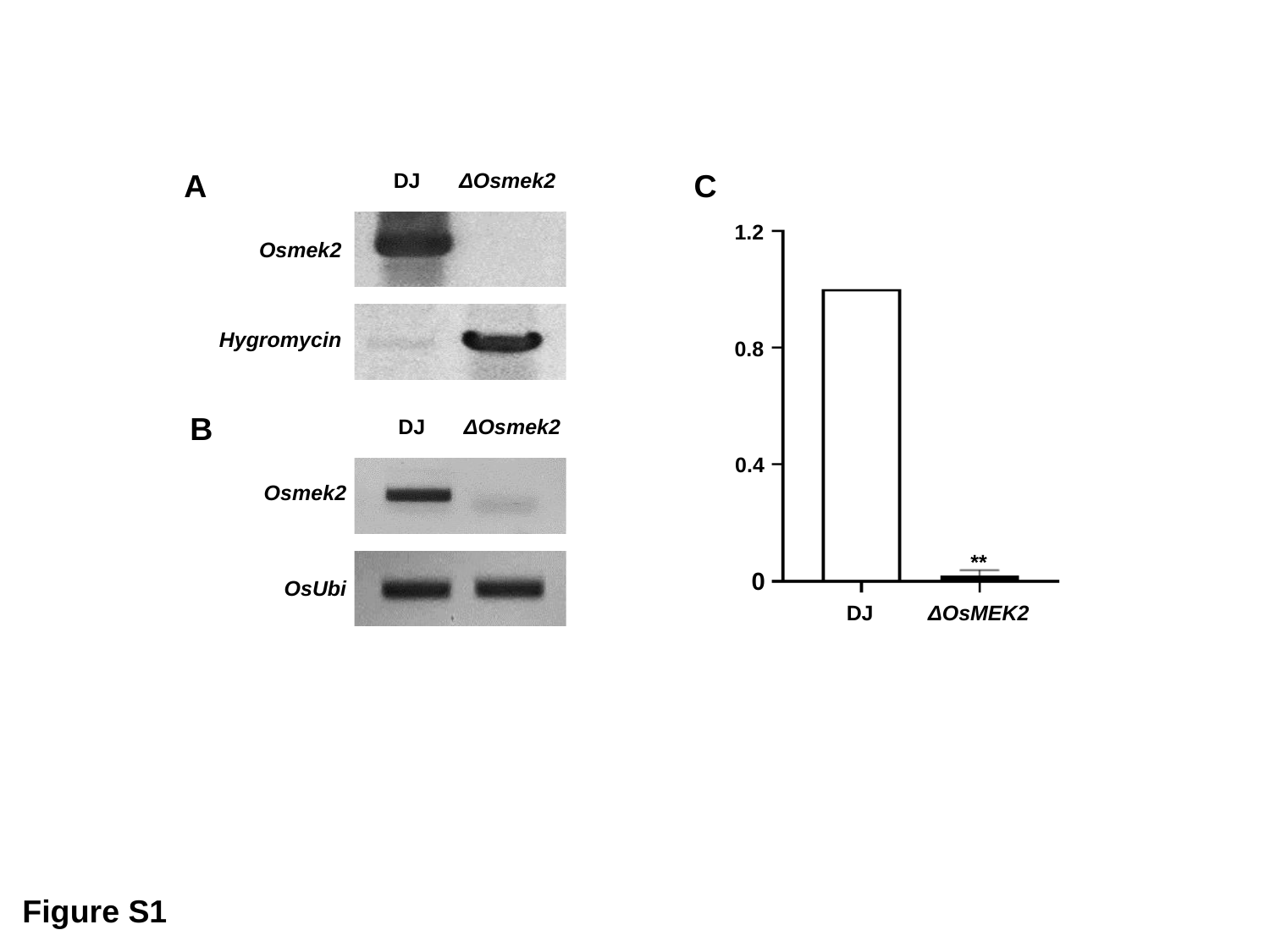

A
C
DJ
ΔOsmek2
Osmek2
Hygromycin
1.2
0.8
B
DJ
ΔOsmek2
0.4
Osmek2
**
0
OsUbi
DJ
ΔOsMEK2
Figure S1

## Slide 2
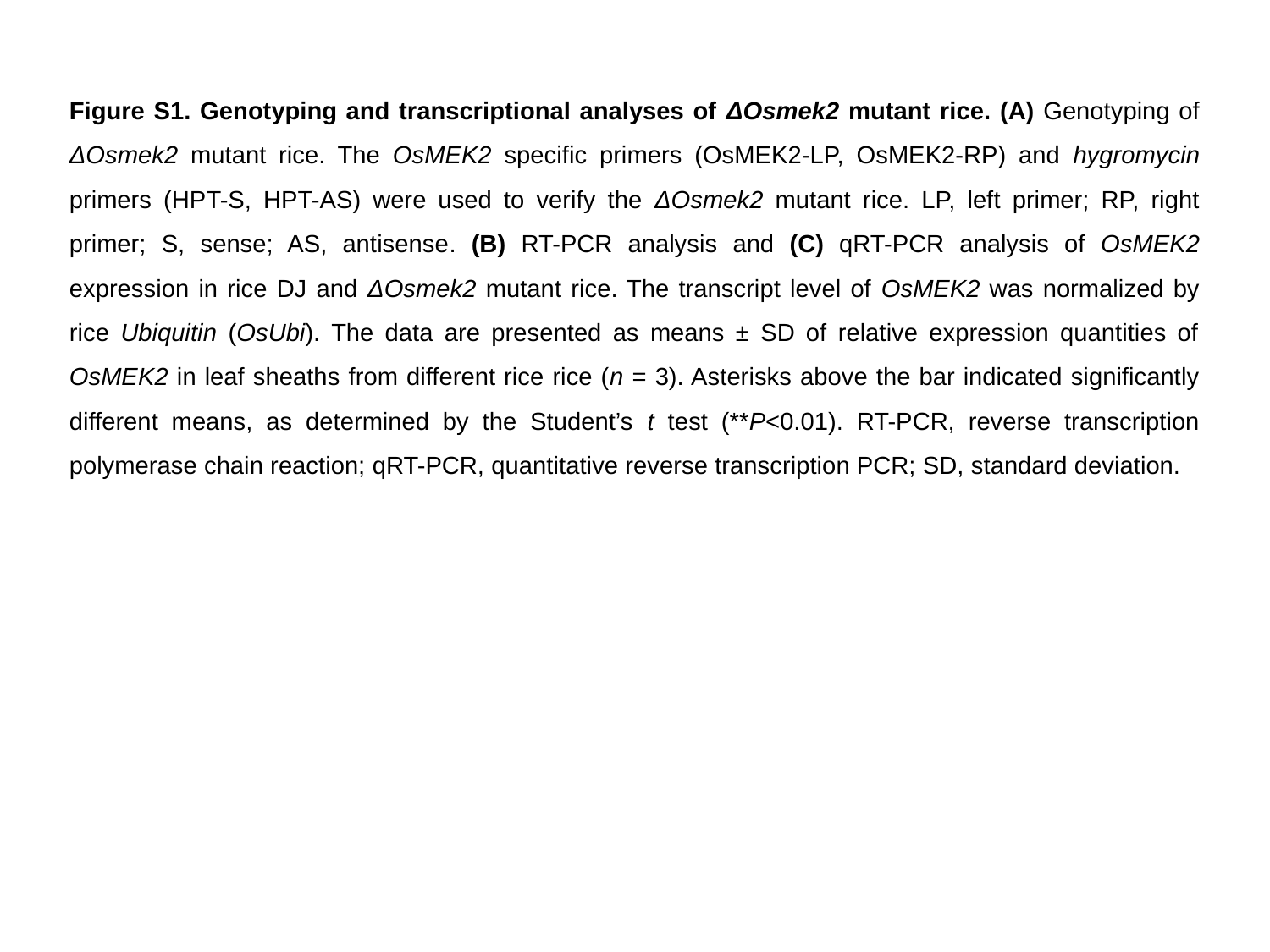

Figure S1. Genotyping and transcriptional analyses of ΔOsmek2 mutant rice. (A) Genotyping of ΔOsmek2 mutant rice. The OsMEK2 specific primers (OsMEK2-LP, OsMEK2-RP) and hygromycin primers (HPT-S, HPT-AS) were used to verify the ΔOsmek2 mutant rice. LP, left primer; RP, right primer; S, sense; AS, antisense. (B) RT-PCR analysis and (C) qRT-PCR analysis of OsMEK2 expression in rice DJ and ΔOsmek2 mutant rice. The transcript level of OsMEK2 was normalized by rice Ubiquitin (OsUbi). The data are presented as means ± SD of relative expression quantities of OsMEK2 in leaf sheaths from different rice rice (n = 3). Asterisks above the bar indicated significantly different means, as determined by the Student’s t test (**P<0.01). RT-PCR, reverse transcription polymerase chain reaction; qRT-PCR, quantitative reverse transcription PCR; SD, standard deviation.

## Slide 3
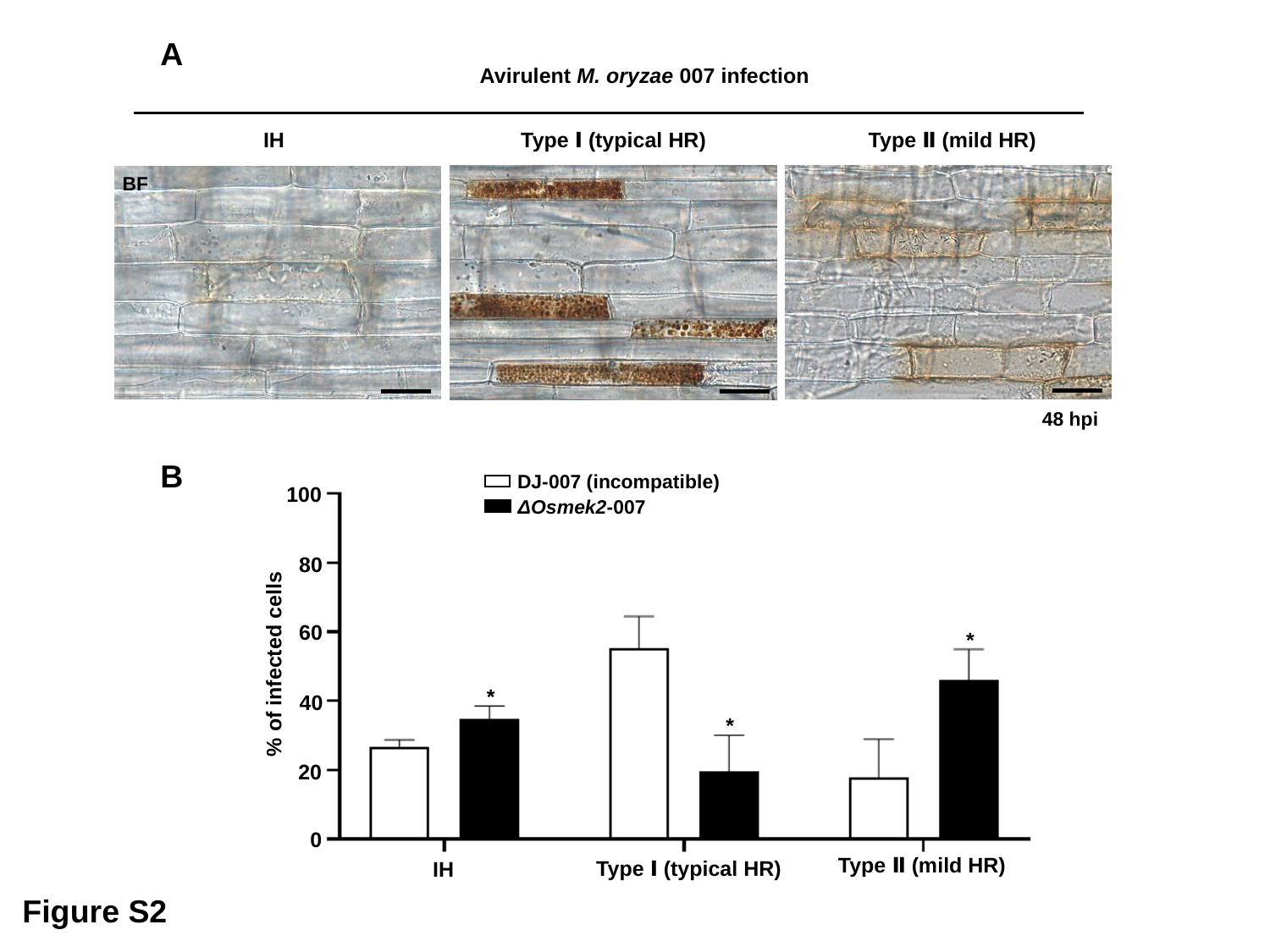

A
Avirulent M. oryzae 007 infection
IH
Type Ⅰ (typical HR)
Type Ⅱ (mild HR)
B
DJ-007 (incompatible)
100
ΔOsmek2-007
80
60
*
% of infected cells
*
40
*
20
0
Type Ⅱ (mild HR)
Type Ⅰ (typical HR)
IH
BF
48 hpi
Figure S2

## Slide 4
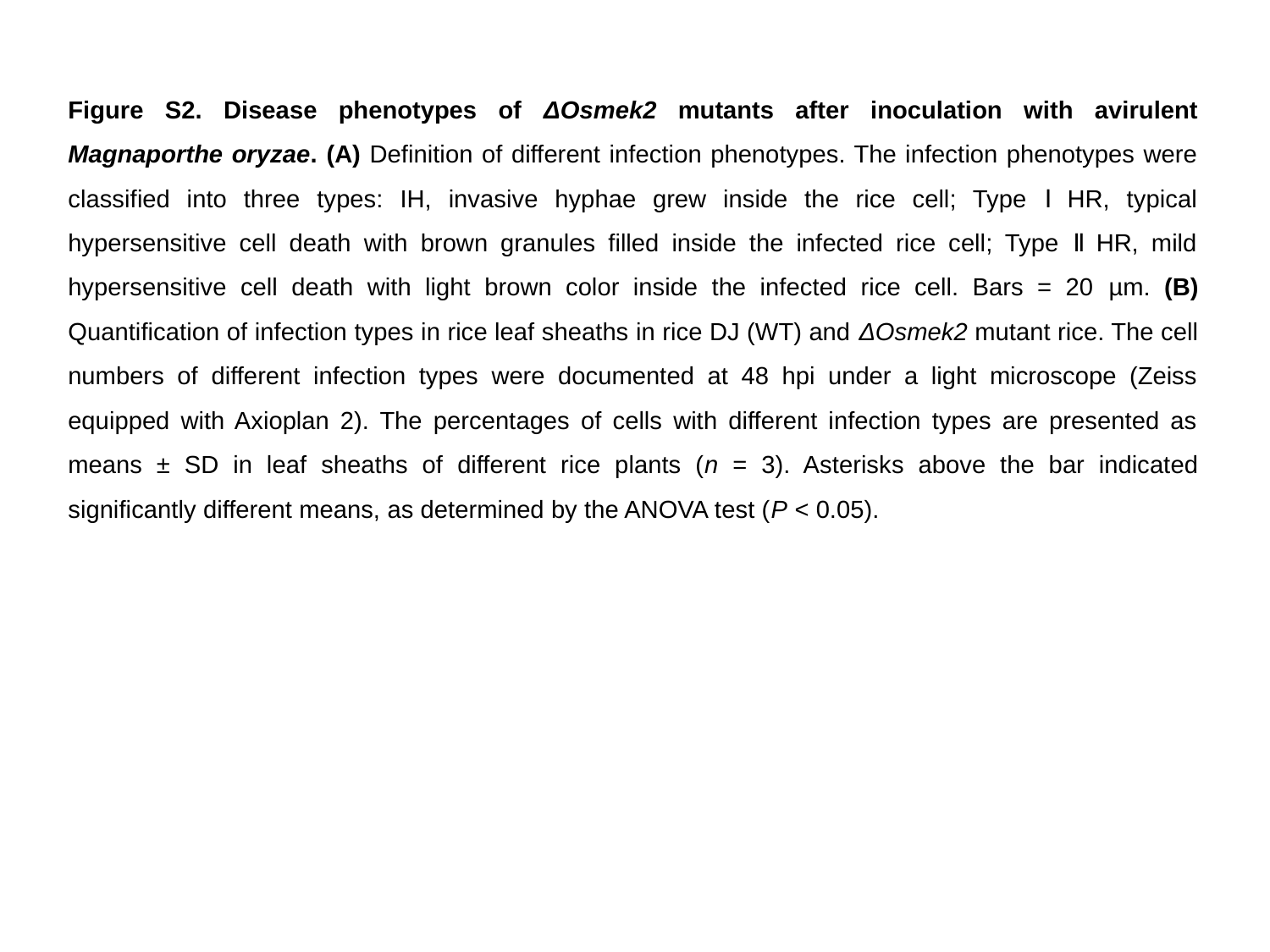

Figure S2. Disease phenotypes of ΔOsmek2 mutants after inoculation with avirulent Magnaporthe oryzae. (A) Definition of different infection phenotypes. The infection phenotypes were classified into three types: IH, invasive hyphae grew inside the rice cell; Type Ⅰ HR, typical hypersensitive cell death with brown granules filled inside the infected rice cell; Type Ⅱ HR, mild hypersensitive cell death with light brown color inside the infected rice cell. Bars = 20 µm. (B) Quantification of infection types in rice leaf sheaths in rice DJ (WT) and ΔOsmek2 mutant rice. The cell numbers of different infection types were documented at 48 hpi under a light microscope (Zeiss equipped with Axioplan 2). The percentages of cells with different infection types are presented as means ± SD in leaf sheaths of different rice plants (n = 3). Asterisks above the bar indicated significantly different means, as determined by the ANOVA test (P < 0.05).

## Slide 5
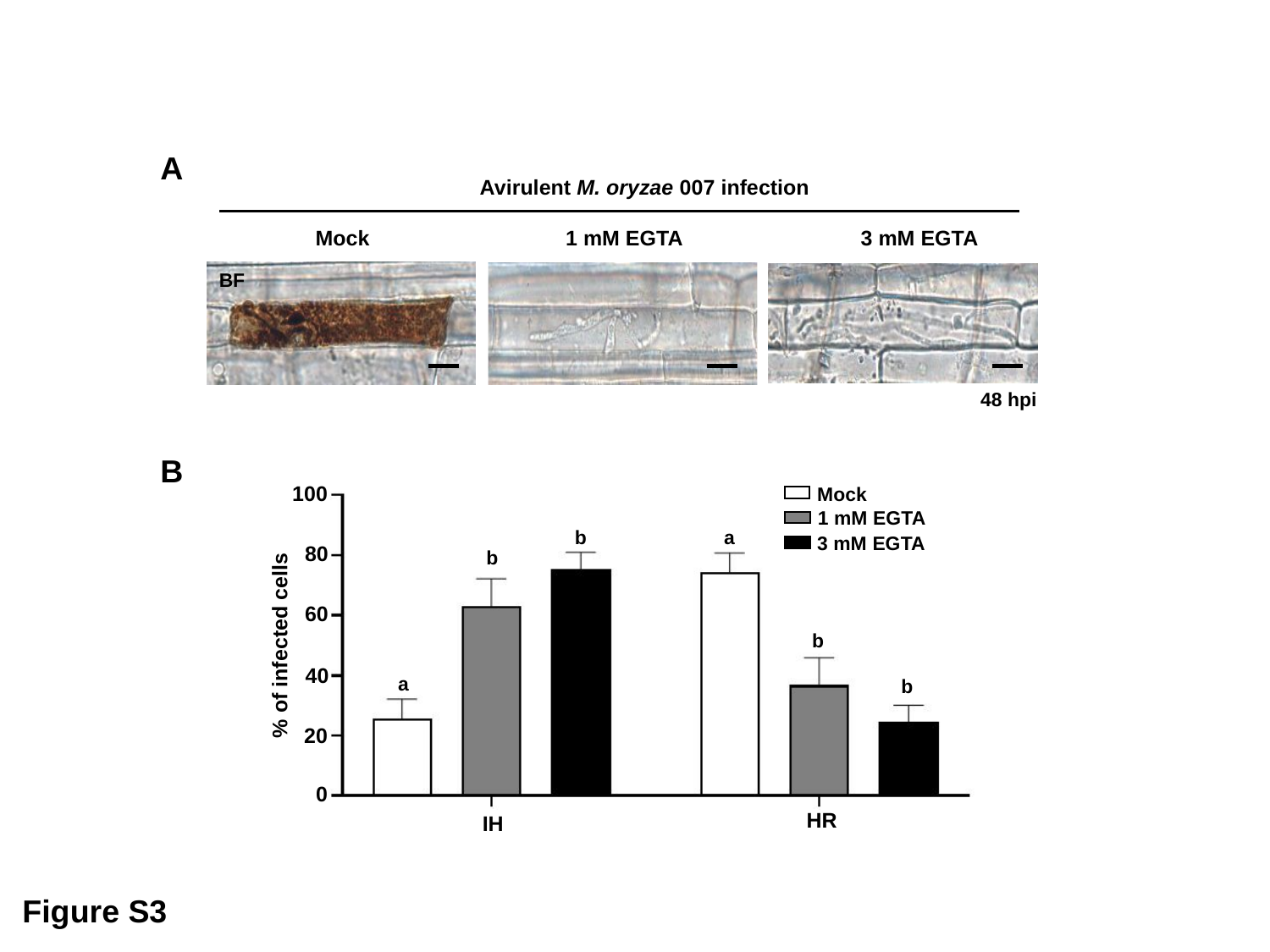

A
Avirulent M. oryzae 007 infection
Mock
1 mM EGTA
3 mM EGTA
BF
48 hpi
B
100
Mock
1 mM EGTA
a
b
3 mM EGTA
80
b
60
b
% of infected cells
40
a
b
20
0
HR
IH
Figure S3

## Slide 6
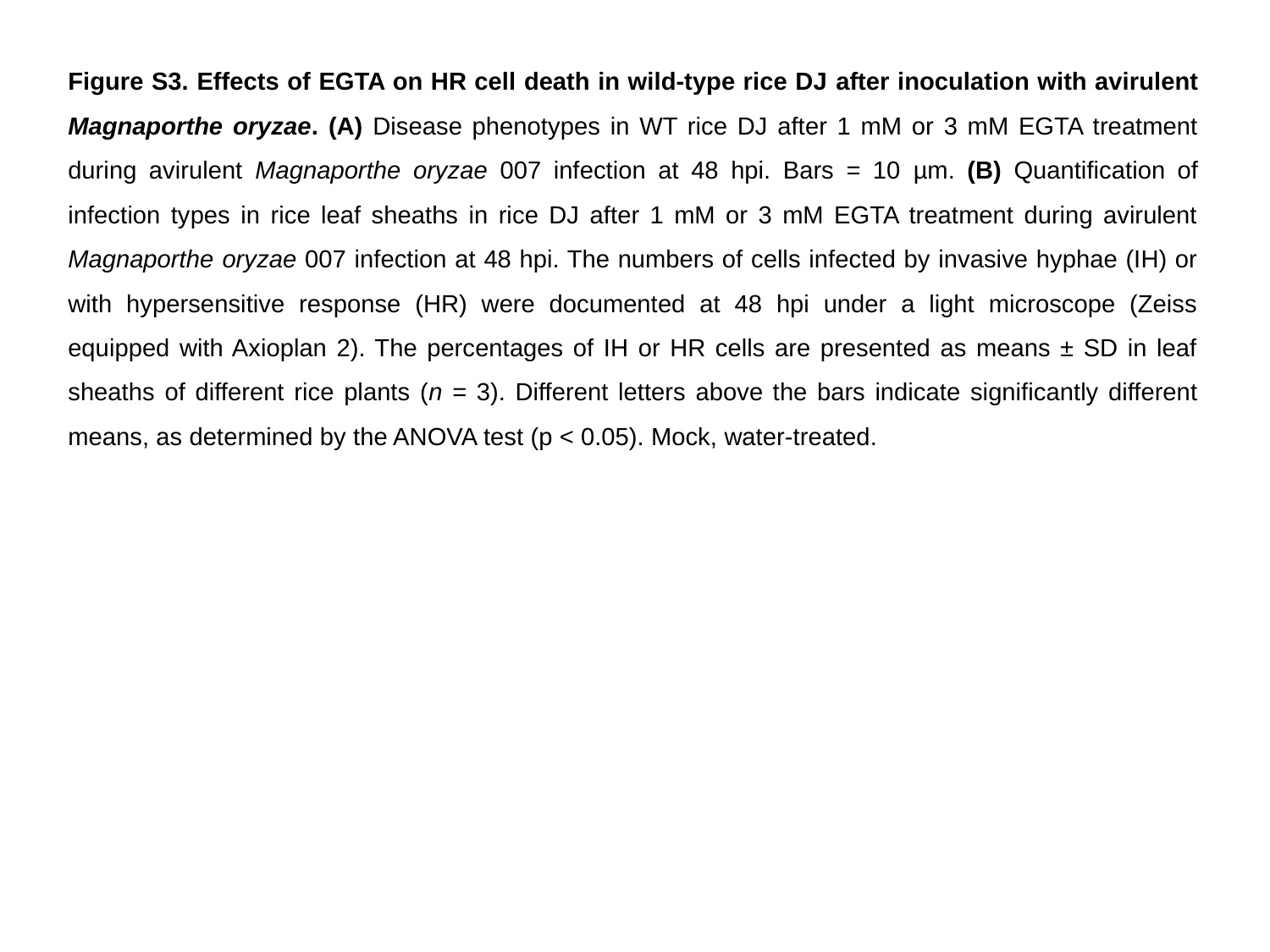

Figure S3. Effects of EGTA on HR cell death in wild-type rice DJ after inoculation with avirulent Magnaporthe oryzae. (A) Disease phenotypes in WT rice DJ after 1 mM or 3 mM EGTA treatment during avirulent Magnaporthe oryzae 007 infection at 48 hpi. Bars = 10 µm. (B) Quantification of infection types in rice leaf sheaths in rice DJ after 1 mM or 3 mM EGTA treatment during avirulent Magnaporthe oryzae 007 infection at 48 hpi. The numbers of cells infected by invasive hyphae (IH) or with hypersensitive response (HR) were documented at 48 hpi under a light microscope (Zeiss equipped with Axioplan 2). The percentages of IH or HR cells are presented as means ± SD in leaf sheaths of different rice plants (n = 3). Different letters above the bars indicate significantly different means, as determined by the ANOVA test (p < 0.05). Mock, water-treated.

## Slide 7
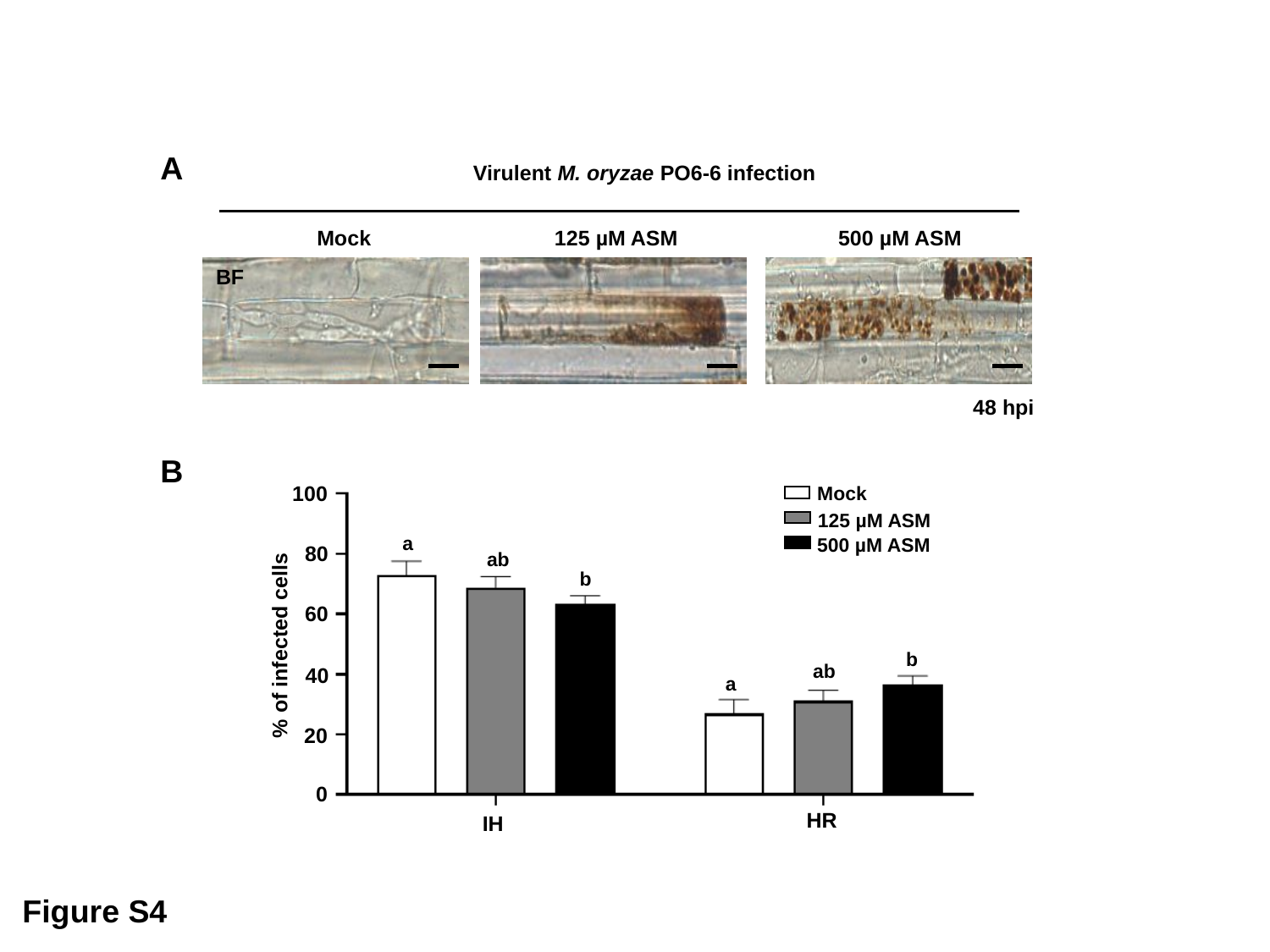

A
Virulent M. oryzae PO6-6 infection
Mock
125 µM ASM
500 µM ASM
BF
48 hpi
B
100
Mock
125 µM ASM
a
500 µM ASM
80
ab
b
60
% of infected cells
b
ab
40
a
20
0
HR
IH
Figure S4

## Slide 8
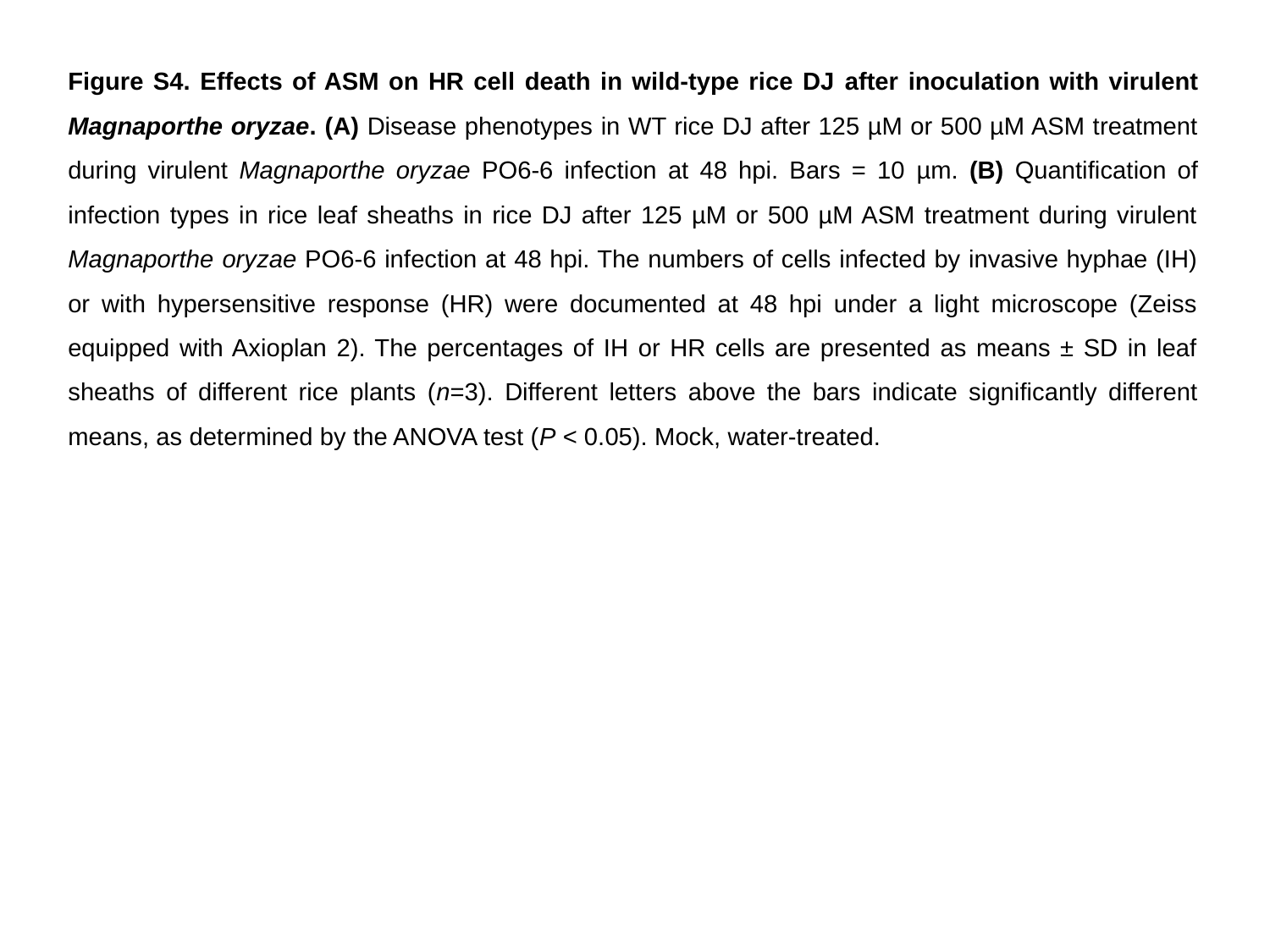

Figure S4. Effects of ASM on HR cell death in wild-type rice DJ after inoculation with virulent Magnaporthe oryzae. (A) Disease phenotypes in WT rice DJ after 125 µM or 500 µM ASM treatment during virulent Magnaporthe oryzae PO6-6 infection at 48 hpi. Bars = 10 µm. (B) Quantification of infection types in rice leaf sheaths in rice DJ after 125 µM or 500 µM ASM treatment during virulent Magnaporthe oryzae PO6-6 infection at 48 hpi. The numbers of cells infected by invasive hyphae (IH) or with hypersensitive response (HR) were documented at 48 hpi under a light microscope (Zeiss equipped with Axioplan 2). The percentages of IH or HR cells are presented as means ± SD in leaf sheaths of different rice plants (n=3). Different letters above the bars indicate significantly different means, as determined by the ANOVA test (P < 0.05). Mock, water-treated.

## Slide 9
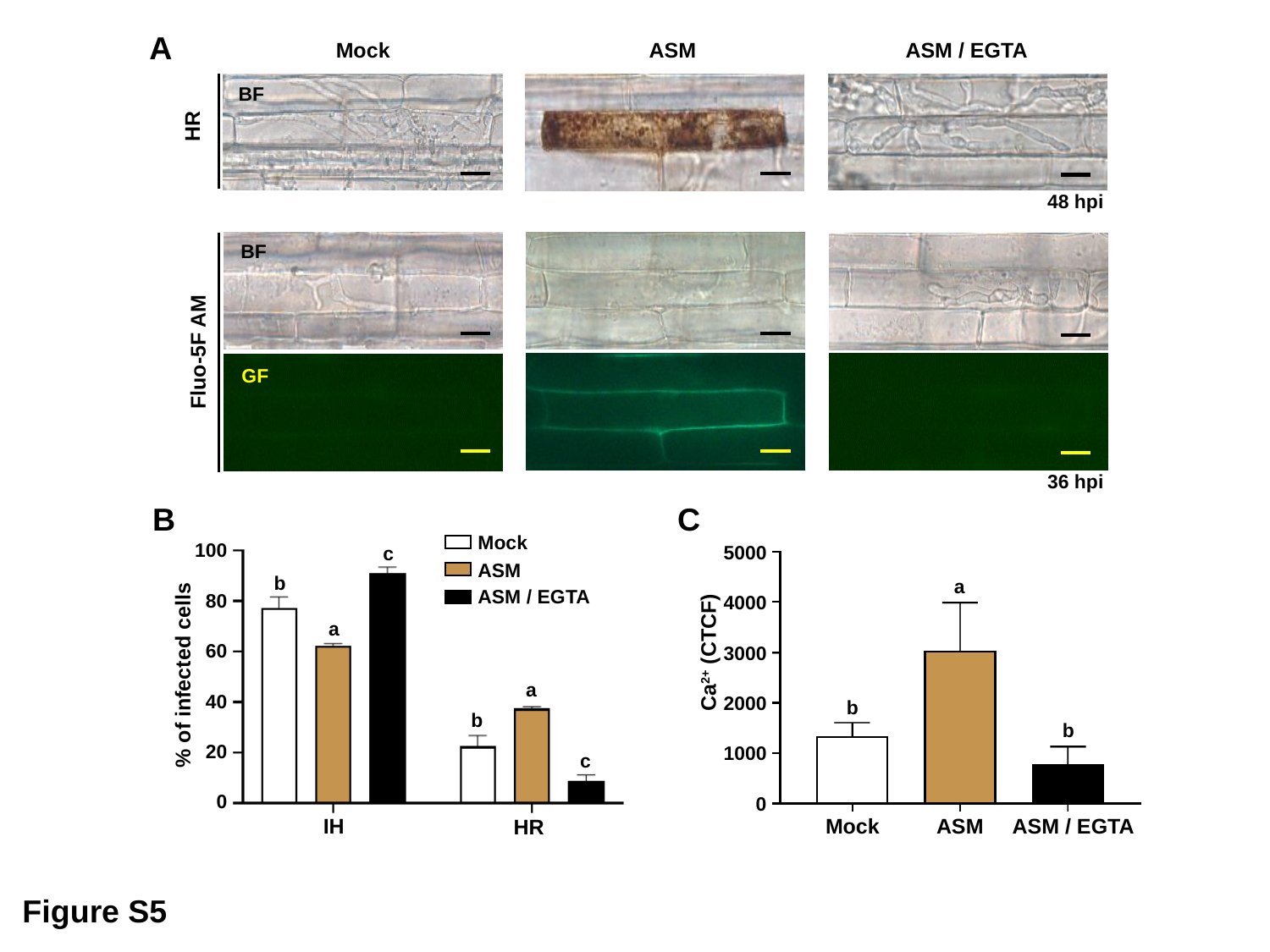

A
Mock
ASM
ASM / EGTA
BF
HR
48 hpi
BF
Fluo-5F AM
GF
36 hpi
B
C
Mock
100
5000
c
ASM
b
a
ASM / EGTA
80
4000
a
Ca2+ (CTCF)
60
3000
% of infected cells
a
40
2000
b
b
b
20
1000
c
0
0
IH
HR
Mock
ASM
ASM / EGTA
Figure S5

## Slide 10
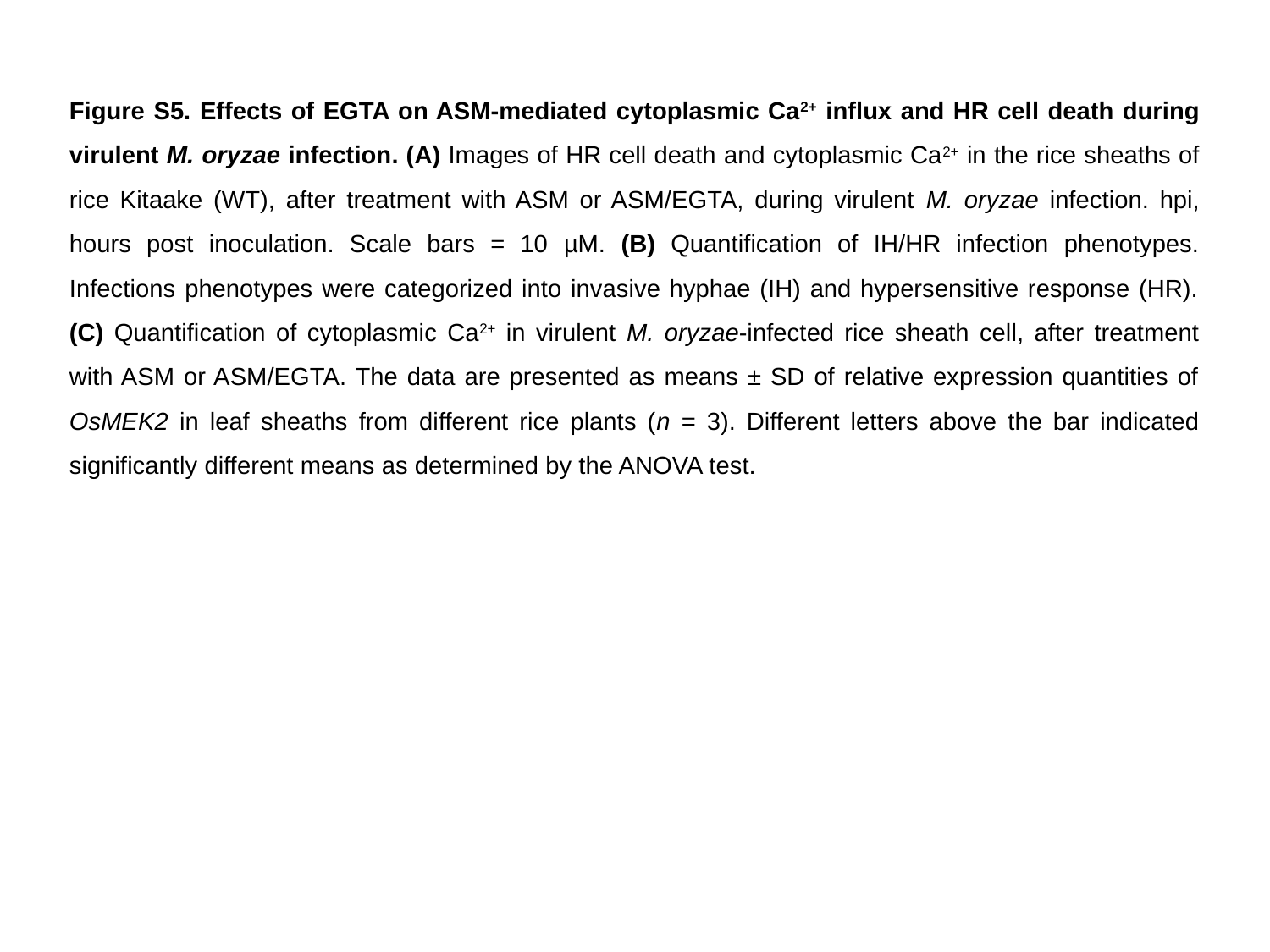

Figure S5. Effects of EGTA on ASM-mediated cytoplasmic Ca2+ influx and HR cell death during virulent M. oryzae infection. (A) Images of HR cell death and cytoplasmic Ca2+ in the rice sheaths of rice Kitaake (WT), after treatment with ASM or ASM/EGTA, during virulent M. oryzae infection. hpi, hours post inoculation. Scale bars = 10 µM. (B) Quantification of IH/HR infection phenotypes. Infections phenotypes were categorized into invasive hyphae (IH) and hypersensitive response (HR). (C) Quantification of cytoplasmic Ca2+ in virulent M. oryzae-infected rice sheath cell, after treatment with ASM or ASM/EGTA. The data are presented as means ± SD of relative expression quantities of OsMEK2 in leaf sheaths from different rice plants (n = 3). Different letters above the bar indicated significantly different means as determined by the ANOVA test.
